# Sugar-Sweetened Beverage Consumption Is Adversely Associated with Childhood Attention Deficit/Hyperactivity Disorder

**DOI:** 10.3390/ijerph13070678

**Published:** 2016-07-04

**Authors:** Ching-Jung Yu, Jung-Chieh Du, Hsien-Chih Chiou, Chun-Cheng Feng, Ming-Yi Chung, Winnie Yang, Ying-Sheue Chen, Ling-Chu Chien, Betau Hwang, Mei-Lien Chen

**Affiliations:** 1 Institute of Environmental and Occupational Health Sciences, School of Medicine, National Yang Ming University, Taipei 11221, Taiwan; d49521001@ym.edu.tw (C.-J.Y.); ryan.feng@gmail.com (C.-C.F.); 2 Department of Pediatrics, Taipei City Hospital, Zhongxiao Branch, Taipei 11556, Taiwan; DAL82@tpech.gov.tw (J.-C.D.); dan75@tpech.gov.tw (B.H.); 3 Department of Child and Adolescent Psychiatry, Taipei City Hospital, Songde Branch, Taipei 11080, Taiwan; DAF28@tpech.gov.tw; 4 Department of Life Sciences and Institute of Genome Sciences, National Yang Ming University, Taipei 11221, Taiwan; mychung@ym.edu.tw; 5 Department of Pediatrics, Taipei City Hospital, Yangming Branch, Taipei 11146, Taiwan; DAH06@tpech.gov.tw; 6 Department of Psychiatry, Taipei Veterans General Hospital, Taipei 11217, Taiwan; drchen3209@gmail.com; 7 School of Public Health, Taipei Medical University, Taipei 11031, Taiwan; lcchien@tmu.edu.tw

**Keywords:** ADHD, sugar-sweetened beverage, artificial food coloring, preservative, case-control, blood lead level, gene polymorphism, children

## Abstract

Attention deficit/hyperactivity disorder (ADHD) is one of the most common childhood neurobehavioral conditions. Evidence of the negative effects of sugar-sweetened beverages (SSBs) on mental health has not been convincing, although a few studies have found an association between high SSB levels and attention problems in children. This study aimed to test the hypothesis that SSB consumption is associated with ADHD among children. Doctor-diagnosed ADHD cases (*n* = 173) and non-ADHD controls (*n* = 159) between age 4 to 15 were recruited. SSB consumption, socio-demographic and lifestyle characteristics of the children, as well as of their mothers’ characteristics during pregnancy, were collected using a questionnaire. Blood lead levels and polymorphisms of two commonly verified dopaminergic-related genes (the D4 dopamine receptor gene DRD4 and the dopamine transporter gene DAT1) were also analyzed. There was a dose-response relationship between SSB consumption and ADHD. After covariates were adjusted, children who consumed SSBs at moderate levels and high levels had 1.36 and 3.69 odds, respectively, of having ADHD, compared with those who did not consume SSBs (*p* for trend < 0.05). Similar results were obtained when females were excluded. Our findings highlighted the adverse correlation between SSB consumption and ADHD and indicated a dose-response effect even after covariates were adjusted.

## 1. Introduction

In recent years, sugar-sweetened beverages (SSBs) have accounted for 80% of the rise in sugar consumption worldwide. Furthermore, SSBs are the largest single source of added sugar consumption [1,2]. SSBs containing high sugar content may cause insulin secretion, drive reactive hypoglycemia and stimulate an increase in epinephrine, which activates hyperactivity disorder behaviors [3]. Additionally, SSBs often contain other additives, such as artificial food colorings (AFCs) and preservatives that could affect children’s behavior [4,5,6,7].

Increasing evidence from animal studies clearly indicates that sugar consumption can cause deficits in cognitive and behavioral functions [8,9,10,11,12]. While many clinical studies on obesity and metabolic disturbances resulting from SSB consumption have been conducted, few studies have examined how SSBs affect development and mental health outcomes [8,13,14,15,16,17,18,19,20,21]. Diet factors, such as sugar consumption, have been linked to an increased risk of attention deficit/hyperactivity disorder (ADHD) [20,22,23]. However, this reported effect is controversial [20,22,23,24,25]. One Norwegian population-based study of 15- to 16-year-old students adopted checklists and questionnaires to assess metal health problems and showed associations between high SSB consumption levels and hyperactivity [20]. A US study of middle school students found that those who consumed SSBs and those who consumed energy drinks had 1.14 and 1.66 odds, respectively, of having ADHD [21]. In a study of disruptive patterns in eating behaviors, Ptacek et al. revealed that male children diagnosed with ADHD exhibited increased sweetened beverage consumption [19]. Their findings suggested that younger children might be susceptible to the adverse effects of SSBs. By contrast, Kim and Chang conducted a Korean study of 107 school-aged children, with only 8.5% categorized as having ADHD, and found no significant association between the consumption of simple sugars, including those in sweets and SSBs, and an increased risk of ADHD [25].

Taiwan has the highest density of chain bubble tea shops and 24-h convenience stores in the world, and both types of stores sell SSBs [26,27]. At the end of 2015, Taiwan had 16,836 bubble tea shops and 10,131 chain convenience stores for a total population of 23.5 million, indicating an average of one store for every 870 people [28]. Researchers have measured the amount of sugar, mainly high-fructose corn syrup (HFCS), added to SSBs and found that it ranged from 22 g to 68 g for a 750 mL serving size [27]. One cross-sectional study evaluated the association between adolescent obesity-related health outcomes and SSB consumption in Taiwan and found that 87.7% of adolescents drank SSBs daily and 25.1% consumed more than 500 mL of SSBs per day [26]. Bubble tea shops have recently arrived in the United Kingdom, Europe, Asia, Australia, Canada and the U.S. [29,30]. Attention should be paid to the intake of these drinks not only because excessive sugar consumption induces metabolic syndrome, which results in excess and imbalanced caloric intake, but also because of its potential association with mental health problems [9,15,20,21,31,32,33], especially among school-aged children.

ADHD is a chronic disease and one of the most common behavioral disorders among children [34,35]. The prevalence of ADHD in children has been reported to range from 7.5% to 9.9% in Taiwan and from 5.9% to 7.1% worldwide [36,37]. ADHD impairs learning and social development; the condition typically develops during the years before school and often persists into adulthood [38,39]. The importance of ADHD prevention and intervention is highlighted by the high prevalence and psychosocial impacts of the disorder. The etiology of ADHD is complex and involves genetic, dietary, and environmental factors that have strong genetic associations, with >70% heritability [40,41,42].

Several studies have assessed the association between SSB consumption and childhood ADHD cross-sectionally [20,21,25]. These studies have measured mental health problems through self-report or interviews with teachers and/or parents. To avoid discordance with the standard nosology of ADHD and prevent misclassification, we ensured that our ADHD cases were diagnosed by doctors based on the Diagnostic and Statistical Manual of Mental Disorders, 4th edition, revised criteria (DSM-IV-TR). Only few doctor-diagnosed case-control studies have been reported; however, these studies have not controlled potential confounding variables [19]. This is the first case-control study to analyze the association between school-aged children’s SSB consumption and ADHD diagnosis by a board-certificated doctor while controlling for covariates. This study hypothesized that children with ADHD drink more SSBs.

## 2. Materials and Methods

### 2.1. Study Participants and Recruitment

The study protocol was approved by the Taipei Veterans General Hospital and Taipei City Hospital institutional review boards. The project identification codes were 97-01-52A, TCHIRB-1010216 and TCHIRB-1030510 (approved on 4 March 2008, 1 July 2012 and 26 August 2014, respectively). Written informed consent was obtained from the participants’ parents or guardians. All participants provided their oral or written assent. This study was conducted in accordance with the revised guidelines of the Declaration of Helsinki. We recruited 4- to 15-year-old subjects from outpatient waiting rooms in both hospitals. In this study, the cases were defined as children with ADHD, as identified by board-certificated pediatricians or psychiatrists after at least a three-visit clinical assessment. Children consecutively admitted for initial and follow-up ADHD treatments were also recruited as cases during the study period. Children with neurological deficits or mental retardation were excluded. The ADHD diagnosis was made in accordance with the DSM-IV-TR criteria [43].

We recruited control subjects by randomly selecting normal 4- to 15-year-old children who visited either of the hospitals for reasons unrelated to ADHD during the same study period, and the same exclusion criteria as those applied to the cases were used. We did not attempt to match cases and controls on gender; however, we matched the two groups on age. Controls were screened for the absence of ADHD symptoms by teachers and parent(s), who assessed children’s behaviors in the classroom and at home, respectively, according to the Chinese version of the Swanson, Nolan and Pelham, Fourth Revision (SNAP-IV) questionnaire [44,45]. The rating results were evaluated by pediatricians to confirm the absence of ADHD symptoms. The SNAP-IV Teacher and Parent Rating Scale, which directly adopted DSM-IV symptoms and used the same format, was translated into Chinese and was found to be a reliable and valid tool for screening for ADHD in clinical and research settings in Taiwan [46,47]. The 26-item SNAP-IV questionnaire was based on a four-point (0–3) rating scale and consisted of the DSM-IV ADHD criteria for inattention (items 1–9) and hyperactivity/impulsivity (items 10–18) as well as criteria for oppositional defiant disorder symptoms (items 19–26). If more than six of the nine inattention or hyperactivity/impulsivity items were given a score of a 2 (quite a bit) or 3 (very much) on either the parent’s or teacher’s form, then the children were defined as at risk of ADHD; otherwise, children were rated as non-ADHD [48]. Children with scores indicating a risk of ADHD were referred to pediatricians or psychiatrists for further confirmation. All cases also received a SNAP-IV score at the initial visit.

We recruited a total of three hundred and thirty-two subjects, one hundred and seventy-three ADHD subjects (*n* = 173) and one hundred and fifty-nine normal controls (*n* = 159). The response rates were 63.6% and 58.9% in the case and control groups, respectively. In examining the association between the intake of SSBs and ADHD, we investigated the following covariates: family factors, maternal lifestyle, participants’ dietary habits, blood lead levels (BLLs) and gene polymorphisms. In this study, participating subjects’ demographic features, dietary habits, mother’s lifestyle during pregnancy, and family history of nervous system diseases were collected via questionnaires. For the measurement of BLLs and analysis of gene polymorphisms, subjects provided a blood (or saliva) sample during the clinic visit.

### 2.2. Dietary Habits, Consumption of SSBs and Intake of Sugar and Calories

We collected information on each child’s dietary habits and SSB consumption. A trained interviewer administered a 30-min questionnaire to the mother or other caretaker. The average weekly consumption of meat (including poultry and livestock), milk, eggs, fish, shellfish and other types of seafood was gathered for the previous month. The questionnaire also included questions that estimated the average weekly consumption of vegetable (light-colored vegetables, such as lettuce, cabbage, and bean sprouts, and dark-colored vegetables, such as spinach and broccoli) and fruit servings. The weekly serving was calculated by multiplying the daily servings with frequency of consumption per week over the previous month. One serving of meat, fish, shellfish or other types of seafood was approximately 35 g (for meat and fish, this was approximately the size of one’s palm). One cup of milk (approximately 250 mL) and one egg or six quail eggs represented a serving of milk and eggs, respectively. One cup of raw leafy vegetables (approximately the size of the participant’s fist) or a half cup of other vegetables represented a serving of vegetables. One serving of fruit was a medium-sized fruit (medium was defined as the size of a baseball) or a half cup of chopped fruit. One serving of SSBs contained 600 mL. Parents or caregivers were asked separate questions about how many servings of milk tea, juice or fruit-flavored drinks, Yakult drinks, and other sugar-sweetened drinks the participants normally consumed. The milk tea referenced in this study is also known as boba milk tea, bubble or pearl tea [49] and is a Taiwanese tea-based drink. It contains a tea base that is mixed/shaken with milk or fruit, and chewy tapioca balls or jellies are often added. Milk tea drinks are part of the larger group of SSB because these beverages are typically HFCS sweetened [50]. Yakult drink is a probiotic dairy product made by fermenting a mixture of milk with a special strain of the bacterium *Lactobacillus casei* Shirota [51]. This beverage is very popular in Taiwan because it contains probiotics and, thus, is likely considered a healthy drink. However, it also contains high content of sugar (approximately 12 grams for every 100 g of Yakult drink) and is classified as a SSB [52]. When questionnaire items were incomplete, we contacted the mothers or caretakers via telephone to obtain the missing information.

Sugar ingested from SSBs was calculated by multiplying the servings by the sugar content in each type of SSB based on a study of the Ministry of Health and Welfare (MOHW) in Taiwan [52]. In addition, the Taiwan Food and Drug Administration (TFDA) Nutrients Database was used to analyze daily caloric intake [53].

### 2.3. Measurement of BLLs and Gene Polymorphism Analysis

Peripheral blood was drawn using a syringe or venoclysis needle and then sealed in a heparin-containing vacuum tube and immediately transported at 4 °C to the laboratory. If individuals were not able to supply a blood sample, DNA was extracted from a saliva sample. Saliva was spat into the Saliva DNA Collection and Preservation Kit (Norgen Biotek Corporation, Thorold, ON, Canada) and stored at room temperature until analysis. The sample preparation and analysis of BLLs and gene polymorphism are described elsewhere [42,54]. The BLLs were measured using inductively coupled plasma-mass spectrometry (Thermo Scientific, Waltham, MA, USA). Trace Elements Serum L-2 (Seronorm™, Billingstad, Norway) was used to verify the precision and accuracy of the analytical measurements. The limit of detection (LOD) for lead was 0.001 μg/dL. Regarding gene polymorphism, 10 tagged SNPs (rs7395429, rs3758653, rs11246228, rs752306, rs6347, rs2975292, rs37022, rs40358, rs10040882, and rs464049) of DRD4 and DAT1 were identified and analyzed.

### 2.4. Covariates

Risk factors potentially associated with ADHD were examined. Predictors were chosen according to their association with ADHD in previous studies. The following variables were considered covariates: gender, body weight, child’s age, maternal age at childbirth, gestational age at birth (<37 weeks or ≥37 weeks), parity (primiparous or multiparous) [55,56], birth order (1st, 2nd and 3rd and above), parent’s education (high school education and below or college or advanced training), maternal history of still or dead birth (yes or no), maternal smoking during pregnancy (yes or no) [57,58,59], maternal alcohol consumption during pregnancy (yes or no) [59,60], and maternal chronic disease during pregnancy (yes or no). In addition to examining these risk factors, we included family history of nervous system diseases in this study. The nervous system diseases listed in the questionnaire included Parkinson’s disease, Alzheimer’s disease, ADHD, mental retardation, cerebral palsy, autism, epilepsy, developmental delay, multiple sclerosis and peripheral neuromuscular disease, and the family members considered for this list were the subjects’ grandparents, parents and siblings. The variables were obtained from clinical records or questionnaires completed by the mothers or caretakers.

### 2.5. Statistical and Probabilistic Analysis

SPSS Version 17.0 (SPSS, Chicago, IL, USA) was used for the statistical analysis. We calculated descriptive statistics based on sample sizes and percentages. Next, we assessed the significance of differences between the case and control groups. A 2-sided nonparametric statistical Mann-Whitney U test was used for consecutive data, and a chi-squared test or Fisher’s exact test was adopted for categorical data when appropriate [61]. Then, we performed logistic regression analysis with ADHD and the consumption of SSBs. Statistical significance was set at *p* < 0.05. The covariates related to ADHD at *p* < 0.05 were controlled in the multivariate analyses. A Kruskal-Wallis test was used to analyze the relationship between ADHD severity and the increased intake of SSBs.

Monte Carlo (MC) simulation, which is a probabilistic analysis, was performed to quantify the theoretical exposure dose range of sugar, AFCs and preservatives. Probability density functions for each parameter were log-normally distributed. Distributions of exposed concentrations and population exposures were estimated by a MC simulation with 5000 replications. Microsoft Excel™ 2010 (Microsoft Inc., Redmond, WA, USA) and Oracle Crystal Ball, Fusion Edition, Release 11.1.2.1.000 software (Oracle Corporation, Redwood City, CA, USA) were used for MC simulation.

## 3. Results

### 3.1. Demographic Characteristics of Participants

Table 1 summarizes the participants’ demographic characteristics and other factors by ADHD classification and the corresponding *p*-values. Among the 332 participants recruited, 173 were diagnosed with ADHD (148 (85.5%) boys and 25 (14.5%) girls) and 159 were normal controls (91 (57.2%) boys and 68 (42.8%) girls). Male ADHD subjects (*n* = 148) markedly outnumbered female ADHD participants (*n* = 25). The age distributions of ADHD and normal controls were similar, with a mean age (±SD) of 8.9 ± 2.0 years and 9.2 ± 2.7 years, respectively (*p* = 0.31). No significant differences in mean age, body weight, child’s age, gestational age at birth, parity, birth order, maternal age at birth, maternal history of still or dead birth, maternal smoking during pregnancy, or maternal chronic disease during pregnancy were found between ADHD patients and controls. As the mother’s and father’s level of education increased, the risk of ADHD decreased (both *p* < 0.01). Compared with the control subjects, ADHD cases showed significant associations with family history of nervous system diseases and maternal alcohol consumption during pregnancy. As displayed in Table S1, mothers with lower education levels smoked significantly more than those with higher education levels (11.5% vs. 3.1%, respectively; *p* < 0.01). Additionally, mothers with lower education levels consumed more alcohol than those with higher education levels, but this association was marginally significant (12.2% vs. 6.2%, respectively; *p* = 0.06).

### 3.2. Dietary Habits

**Figure 1:**
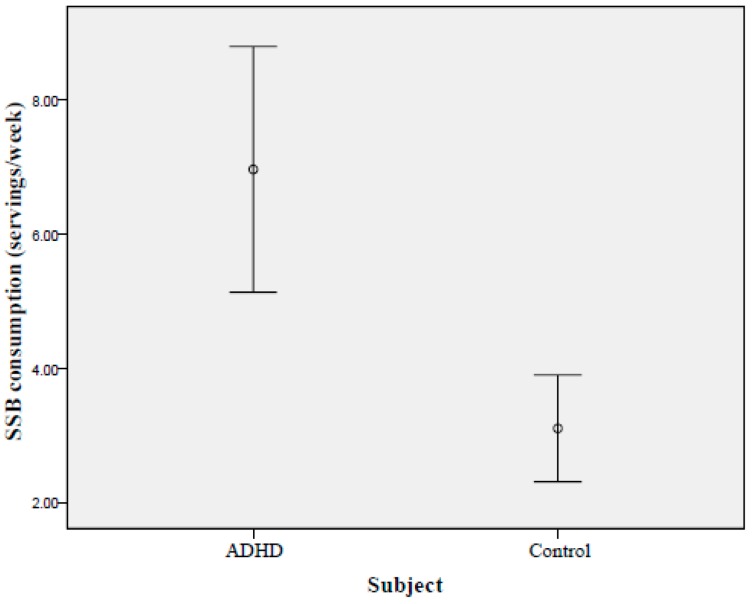
Mean consumption (95% CIs) of SSBs among controls and children with ADHD (unit = servings/week).

As shown in Table 2, approximately half of the children in both the control and ADHD groups consumed at least one serving of SSBs each week (56.0% and 48.5%, respectively). ADHD children consumed significantly more servings of SSBs than normal controls (6.96 ± 9.27 vs. 3.10 ± 5.08 servings/week, respectively; *p* < 0.01). Figure 1 presents the mean and 95% CI of SSB consumption and shows higher levels of SSB consumption among ADHD cases than among controls. ADHD children also ingested significantly more meat and milk (*p* = 0.04 and *p* = 0.02, respectively). Control subjects’ intake of vegetables and fruits was significantly higher than that of children with ADHD (*p* < 0.01 and *p* = 0.02, respectively). No difference was observed in the consumption of eggs, fish and other types of seafood between the two groups.

### 3.3. Sugar Consumption and Caloric Intake

The levels of sugar intake from SSBs and daily caloric intakes of the ADHD and normal control groups are presented in Table 2. The MOHW in Taiwan reported that the average dietary reference intake (DRI) of children 6- to 12-years-old was 2008 calories, with 2113 calories for boys and 1904 calories for girls [62]. ADHD children’s total caloric intake was significantly higher than that of the control subjects (2061.1 ± 689.7 vs. 1801.6 ± 448.3 calories/day, respectively; *p* < 0.01), representing 102.6% and 89.7% of their DRI, respectively. In addition, the daily caloric intakes of male and female ADHD subjects were significantly higher than those of their counterparts in the control group (*p* = 0.04 and *p* < 0.01, respectively). We found significant differences between these two groups in energy intake per day attributed to the SSB consumption (327.2 ± 440.0 vs. 141.4 ± 225.2 calories/day, respectively; *p* < 0.01); the same observations were achieved for both boys and girls (*p* < 0.01 and *p* = 0.02, respectively). For children with and without ADHD, the average daily calories obtained from SSBs were 15.9% and 7.8%, respectively. The levels of sugar intake from SSBs of the ADHD group were significantly higher than those of the control group (296.7 ± 419.5 vs. 117.3 ± 198.1 g/day, respectively; *p* < 0.01).

### 3.4. The Association between SSB Consumption and ADHD

The crude and adjusted odds ratios (ORs) and their confidence intervals (CIs) from the bivariate analysis and multivariate analysis are shown in Table 3. The results showed a dose-response relationship between ADHD and SSB consumption. The risk of ADHD increased by 48% (OR = 1.48, 95% CI: 0.79–2.78) for children who consumed 1–6 servings/week, and the OR was 4.66 (95% CI: 2.15–10.09) for children who consumed ≥7 servings/week. These values were all significantly higher than those for children who did not consume SSBs (*p* for trend < 0.01). The adjusted OR showed that children who consumed 7 or more SSB servings per week had a nearly 4-fold greater odds of having an ADHD diagnosis than the reference group after other variables were taken into account (OR: 3.76, 95% CI: 1.31–10.80, *p* for trend of 0.02). We observed that children with higher SSB consumption had a greater risk of having ADHD.

Boys and girls reported similar SSB consumption (4.75 ± 6.90 vs. 4.29 ± 7.92 servings/week; data not shown). Because of the limited number of females with ADHD in this study (*n* = 25), a sensitivity analysis was performed by excluding females. A logistic regression analysis excluding females suggested that boys with ≥7 servings/week of SSB intake had a greater risk of having an ADHD diagnosis than the reference group (Table 3). The ORs were essentially the same after covariates were adjusted (*p* for trend of 0.06). A marginally significant dose-response relationship was also observed between ADHD and the consumption of SSB for boys after gender, consumption of milk/meat/fruit/vegetables, family history of nervous system diseases, parental education levels, maternal alcohol consumption during pregnancy, and gene polymorphism of DRD4 at rs752306 were controlled. 

### 3.5. Gene Polymorphisms

Table S2 lists the 10 SNPs that were related to genetic variations in DRD4 and DAT1 between participants. Dopaminergic gene variations among children were evaluated as potential confounders in our study. Genetic variations of DRD4 in rs752306 were related to ADHD at *p* < 0.05 and were controlled in the regression model.

### 3.6. Lead Exposure

The collection of blood samples during participant recruitment, especially among school-aged ADHD participants, was challenging. We collected 151 blood samples (46 cases and 105 controls). The BLLs were detected in 100% of the participants because LOD was low (i.e., 0.001 µg/dL) and ranged from 0.44 to 4.71 µg/dL. The mean (±SD) of BLLs among the control and ADHD participants was similar (1.75 ± 0.77 µg/dL and 1.62 ± 0.79 µg/dL, respectively, *p* = 0.15). No significant difference in BLLs was found between children with and without ADHD (Figure S1). Therefore, blood sample collection was terminated.

## 4. Discussion

### 4.1. The Association Between SSB Consumption and ADHD

In this case-control study, we reported that SSB consumption was associated with doctor-diagnosed ADHD. After other variables were controlled, children with greater SSB consumption had a nearly 4-fold greater odds ratio of having an ADHD diagnosis compared with those who consumed fewer SSBs, indicating a dose-response effect. Moreover, the logistic regression analysis without females suggested that males with higher SSB consumption levels were at greater risk of having ADHD, with a statistically marginal dose-response effect (*p* for trend is 0.06), compared with males in the control group (Table 3). This is the first case-control study to investigate the association between children’s SSB consumption and ADHD diagnosis by a board-certified doctor while controlling for covariates.

Our findings are consistent with those of the limited studies that analyzed the association between SSBs and ADHD. Schwartz et al. conducted a health behavior survey and hyperactivity/inattention questionnaire on 1649 U.S. children and found that greater SSB consumption was associated with an increased risk of having ADHD [21]. A dose-response relationship between soft drink consumption and hyperactivity, as measured with a questionnaire, was found among Norwegian students even after possible cofounders were adjusted [20]. Although it did not adjust for covariates, another case-control study of 6- to 10-year-old Czech males with and without ADHD found that disruptive patterns in eating habits among children with ADHD were associated with increased consumption of SSBs [19].

The analysis of SSBs conducted in this study mainly focused on milk tea, juice or fruit-flavored drinks and Yakult drinks, which are the most popular and frequently studied SSBs in Taiwan [26,27,49,50]. The component of SSBs was gathered from the information labeled on the bottle or listed on the menu/instructions [52]. SSBs’ effect on childhood ADHD may be due to the effects of sugar, AFCs, and preservatives, which are substances found in SSBs and in other foods and products. Dietary patterns established in childhood may continue into adulthood [63,64]. The type of foods sold or served in schools is an important environmental factor that contributes to children’s dietary patterns, and the government should limit the availability of SSBs in schools to promote healthy eating behaviors among youth [65].

### 4.2. Effect of Sugar in SSBs on ADHD

Several previous studies have suggested an adverse relationship between sugar consumption (not specifically SSBs) and ADHD [7,22,66,67]. By contrast, a meta-analysis performed in the mid-1990s and mid-2010s concluded that sugar was not a proven risk factor of hyperactivity among children [18,68]. Sugar consumption is believed to cause behavioral problems because it causes: (1) sugar intolerance (discomfort experienced after eating sugar or sugary foods); (2) reactive hypoglycemia following ingestion; and (3) a reduced intake of essential micronutrients [69]. Research has suggested that the adverse effects of sugar may be due to ADHD children’s inclination to consume more SSBs, but many studies have also reported the converse relationship [67,69,70]. Animal studies have also shown that prenatal sucrose consumption is a risk factor for ADHD, but this association may need to be further investigated among humans [71]. The total sugar content of popular US beverages ranged from 5.5 to 12.7 g per 100 mL [72]. The sugar content of SSBs in Taiwan was similar to that of SSBs in the U.S., ranging from 7.3 to 11.9 g per 100 mL [52]. ADHD children’s sugar consumption from SSBs was significantly higher than that of control subjects (296.7 ± 419.5 vs. 117.3 ± 198.1 g/week, respectively; *p* < 0.01). Because SSBs are the principle source of added sugar in diets, SSB consumption may be associated with a concurrent reduction in essential nutrient intake, hypoglycemia and sugar tolerance [69,73,74]. Our study found a positive relationship between SSB intake and ADHD. Thus, public health efforts to promote healthy beverage choices and decrease SSB consumption should be actively employed.

### 4.3. Effects of Artificial Food Colorings (AFCs) in SSBs on ADHD

SSBs often contain other additives that could affect behavior, such as food coloring and preservatives. Some studies have shown an adverse association between AFCs and hyperactivity among children, but other results have been contradictory [6,75,76,77]. Major concerns regarding AFCs have arisen due to the composition of azo dye [78]. The top three AFCs used in food are allura red, tartrazine, and sunset yellow, representing 90% of the AFCs used [78]. The maximum allowable concentration of these three AFCs is 0.10 g/kg [4,79]. In this study, the 95th percentile AFC exposure levels for ADHD and control children were simulated by MC because this percentile was assumed to represent the worst case scenario. The estimated maximum daily AFC exposure was calculated as follows [80]:

Estimated maximum daily exposure of AFCs (mg/day/kg b.w.) = 95th percentile from MC simulation of levels of SSB consumption (servings/day) × volume of one serving (kg/serving) × maximum allowed concentration of AFCs (mg/kg)/children’s body weight (30 kg) × percentage of beverages with AFCs (31%)(1)

The margin of safety (MOS) used for the health risk assessment in Table S3 was calculated by dividing the ADI by the estimated maximum daily exposure. The MOSs for the worst case scenario among ADHD and control groups were all greater than 1, indicating no concern of incurring any appreciable health risk. Our estimated exposure to AFCs, 0.97–1.93 mg/day/kg b.w. is in line with those found in several studies. In Germany, although the SSBs that children consumed contained different colors, the mean intake of AFCs was 0.16–0.50 mg/day/kg b.w. [80]. In an Irish survey, the mean intake of AFCs was 0.39–2.77 mg/day/kg b.w., which was well below the ADI for children. For Australian children aged 6–12, the dietary exposure to AFCs was also below the ADI, even among those in the 90th percentile exposure level [81]. In the past 50 years, the daily consumption of AFCs per capita has increased 4-fold [4]. Although the exposure to AFCs reported in these studies was below the ADI, we suggest that children’s exposure to AFCs should be minimized until the safety of AFCs can be refined.

### 4.4. Effect of Preservatives in SSBs on ADHD

Preservatives in children’s food and beverages have also been reported to influence their hyperactive behavior and ADHD symptoms [5,76,82,83]. Benzoic acid (BA) and sorbic acid (SA) are often added to drinks as preservatives in the form of sodium or potassium salts [84,85], and they have a maximum acceptable beverage concentration of 1.0 g/kg [53]. The maximum permitted amount of BA and SA in SSBs is 1.0 g/kg. Thus, the 95th percentile of preservative exposure from SSBs was 62.40 mg/day/kg b.w. for ADHD children and 31.54 mg/day/kg b.w. for control children. In the worst case scenario, the MOSs for the ADHD and control groups were smaller than 1, which should be of great concern (Table S4). In our study, the estimated maximum exposure of BA and SA in the worst case scenario was much higher than that found in studies conducted in other nations [5,77]. Therefore, to raise awareness of preservative exposure through SSBs and its health implications, the allowable preservative concentration in SSBs should be lowered.

### 4.5. The Relationship between Dietary Habits and SSBs

We found that high maternal and paternal levels of education were associated with a decreased risk of ADHD (Table 1). This finding is in line with previous results indicating that low parental education levels are positively associated with ADHD [86,87,88]. The higher parental level of education among the control group may confer economic advantages, such as healthier meals. Therefore, these children ingest more vegetables and fruits [89,90]. Our study is consistent with the 2011 study conducted by Howard that revealed that an “unhealthy”, Western-style diet (i.e., more meat and sweets and fewer vegetables and fruits) was associated with ADHD [91]. Families with high educational levels likely pay more attention to the child’s alimentation, and such attention may play a protective role in children’s neurodevelopment [24]. On the contrary, parents with low educational levels have been shown to be less organized in terms of alimentation habits [92]. Their children might also engage in less organized activities, cognitive tasks and cultural communication, potentially increasing these children’s risk of developmental problems [93].

The caloric intakes were 102.6% and 89.7% of the DRI for children with and without ADHD, respectively. In this study, children without ADHD seemed to consume fewer calories than the DRI. Although the potential causal link between diet and ADHD is controversial, nutritional education programs for patients and/or their families should be considered.

The World Health Organization (WHO) has proposed that the proportion of daily calories obtained from sugar should be 10% or less [25]. In Taiwan, the average percentage of caloric intake from SSBs among school-aged children was 7.1% [62], which is consistent with our reported intake among children in the control group (7.9%). However, in our study, ADHD children consumed 15.9% of their daily calories from SSBs, which exceeds the recommended levels of the WHO and is quite concerning. In Taiwan, according to the new nutrition labeling requirement launched on July 1st of 2015 [94], sugar content should be labeled on packaged foods. Therefore, children, especially those with ADHD, should receive further education to inform them of the nutrition information on the label, to aid their drink selection, and to prevent their unintended overindulgence of sugar.

### 4.6. Children’s Exposure to Lead in Taiwan

Low-level lead exposure, even at concentrations much lower than the previous action limit of 10 µg/dL (currently tightened to 5 µg/dL), has been associated with the clinical diagnosis of ADHD in several recent studies [95,96,97]. We observed no significant difference in BLLs between the two groups (1.75 ± 0.77 µg/dL and 1.62 ± 0.79 µg/dL, respectively; *p* = 0.15). The BLLs in this study were lower than those reported in the other two Taiwanese studies [98,99]. Since 2000, leaded gasoline has been banned in Taiwan. Additionally, 97.1% of the participants in the present study lived in the Greater Taipei area, which has low pollution levels and tight control of industrial emissions. The above reasons may explain the lower BLL measurements in our study compared with those in studies performed in 2002 and 2012.

### 4.7. Strengths and Limitations

This study has a number of strengths. This is the first case-control study to evaluate the association between SSB consumption and ADHD among children while adjusting for covariates. Moreover, the SNAP-IV questionnaire, a tool with acceptable reliability, was used to screen children suspected to have ADHD. Patients with an ADHD diagnoses were confirmed through extensive evaluations based on the DSM-IV-TR and performed by pediatricians or psychiatrists. This made our confirmation of ADHD cases more stringent than studies that used an interview with a student, parent, or teacher to ascertain ADHD diagnostic status [20,25,67]. Thus, the likelihood of misclassification between the ADHD and non-ADHD groups was minimal. We were also able to examine and control for known possible confounders, including dopamine-related gene polymorphisms, lead exposure, several socioeconomic indicators, and mother and child lifestyle factors, in our multivariate analyses.

However, this study also has limitations. Recall bias of participant characteristics, such as prenatal tobacco and alcohol exposure and family history of nervous diseases, likely impacted our study. We attempted to minimize recall bias by requesting information that did not heavily depend on memory or subjective interpretation, such as parental education level and maternal age at birth. Additionally, because this study did not select participants based on SSB consumption, the likelihood of selection bias was low. Another limitation is that this study did not conduct experiments on the relationship between increased intake of SSBs and the severity of ADHD symptoms. Whether increased intake of SSBs worsens ADHD symptoms or restricted intake of such beverages could reduce ADHD symptoms remain unknown. Moreover, due to quantification difficulties, we did not include the consumption of other foods or processed foods that may contain sugar, AFCs and preservatives, such as cookies, candy and sweets, in the questionnaire. Furthermore, foods with additives, such as monosodium glutamate (MSG), which might increase the risk of having ADHD, were not included in the questionnaire. Considerable clinical and experimental evidence suggests that deficiencies or imbalances in certain micronutrients, such as vitamins A, C, D, B6 and minerals may contribute to ADHD [24,100,101,102]. However, information on micronutrient status is not available in this study. Because the subjects in this study live in the municipal greater Taipei area, the likelihood that they consume insufficient micronutrients is minimal. Further studies on the relationship between micronutrient status and ADHD are needed in Taiwan.

## 5. Conclusions

In this case-control study of Taiwanese children, a dose-response relationship was found between SSB intake and ADHD. This association remained unchanged after gender; consumption of milk, meat, fruits, and vegetables; parental education level; maternal alcohol consumption during pregnancy; family history of nervous system disease; and DRD4 genetic variations in rs752306 were controlled. In this case-control study, we have shown an association between SSB consumption and ADHD. However, we cannot demonstrate causality. The consumption of SSBs might be a consequence rather than a cause of ADHD. Future research should explore the direction of and the mechanisms underlying this association.

## Figures and Tables

**Table 1:** Demographic characteristics of the study participants (*N* = 332).

Variables	Controls	ADHD	*p*-Value
	*n* = 159	*n* = 173	
**Demographic characteristics**			
**Gender (%)**			<0.01 *
Female	68 (42.8%)	25 (14.5%)	
Male	91 (57.2%)	148 (85.5%)	
**Body weight**	32.1 ± 11.6	33.8 ± 12.3	0.20
**Age (years)**	8.9 ± 2.0	9.2 ± 2.7	0.31
**Gestational age at birth (%)**			0.34
<37 weeks	15 (9.4%)	22 (12.7%)	
≥37 weeks	144 (90.6%)	151 (87.3%)	
**Parity (%)**			0.80
Primiparous	87 (54.7%)	97 (56.1%)	
Multiparous	72 (45.3%)	76 (43.9%)	
**Birth Order (%)**			0.87
1st	87 (54.7%)	93 (54.7%)	
2nd	56 (35.2%)	57 (33.5%)	
3rd and above	16 (10.1%)	20 (11.8%)	
**Paternal education level (%)**			<0.01 *
High school or below	39 (24.5%)	98 (56.6%)	
College or advanced training	120 (75.5%)	75 (43.4%)	
**Maternal education level (%)**			<0.01 *
High school or below	46 (28.9%)	93 (53.8%)	
College or advanced training	113 (71.1%)	80 (46.2%)	
**Family history of nervous system diseases (%)**			0.02 *
No	130 (81.8%)	123 (71.1%)	
Yes	29 (18.2%)	50 (28.9%)	
**Maternal age at birth**	30.3 ± 4.4	30.1 ± 5.0	0.80
**History of still or dead birth (%)**			0.17
No	117 (73.6%)	115 (66.5%)	
Yes	42 (26.4%)	58 (33.5%)	
**Smoking during pregnancy (%)**			0.50
No	150 (94.3%)	160 (92.5%)	
Yes	9 (5.7%)	13 (7.5%)	
**Alcohol consumption during pregnancy (%)**			<0.01 *
No	152 (95.6%)	151 (87.3%)	
Yes	7 (4.4%)	22 (12.7%)	
**Chronic disease during pregnancy (%)**			0.16
No	147 (92.5%)	152 (87.9%)	
Yes	12 (7.5%)	21 (12.1%)	

* p < 0.05.

**Table 2:** Dietary characteristics of the study participants (*N* = 260).

Variables	Controls	ADHD	*p*-Value
	*n* = 159	*n* = 101	
**Dietary habits**			
**Total SSBs consumed (servings/week)**			<0.01 *
	3.10 ± 5.08	6.96 ± 9.27	
**Categorized SSB consumption (servings/week)**			<0.01 *
0	51 (32.1%)	19 (18.8%)	
1–6	89 (56.0%)	49 (48.5%)	
≥7	19 (11.9%)	33 (32.7%)	
**Milk tea (%)**			<0.01 *
0	104 (65.4%)	52 (51.5%)	
1–6	51 (32.1%)	37 (36.6%)	
≥7	4 (2.5%)	12 (11.9%)	
**Juice or fruit-flavored drinks**			<0.01 *
0	109 (68.6%)	50 (49.5%)	
1–6	49 (30.8%)	45 (44.6%)	
≥7	1 (0.6%)	6 (5.9%)	
**Yakult drinks**			<0.01 *
0	108 (67.9%)	49 (48.5%)	
1–6	50 (31.4%)	47 (46.5%)	
≥7	1 (0.6%)	5 (5.0%)	
**Other SSBs**			0.11
0	91 (57.2%)	45 (44.6%)	
1–6	63 (39.6%)	50 (49.5%)	
≥7	5 (3.1%)	6 (5.9%)	
**Rice/Noodle (servings/week)**	20.53 ± 5.30	20.42 ± 4.62	0.34
**Meat (servings/week)**	14.02 ± 9.98	18.14 ± 13.89	0.04 *
**Milk (servings/week)**	3.33 ± 2.93	5.01 ± 5.02	0.02 *
**Eggs (servings/week)**	4.32 ± 2.78	4.43 ± 3.01	0.77
**Vegetables (servings/week)**	16.61 ± 12.64	11.97 ± 9.04	<0.01 *
**Fruits (servings/week)**	5.70 ± 3.61	4.78 ± 3.68	0.02 *
**Fish (servings/week)**	5.92 ± 7.74	6.66 ± 8.22	0.65
**Shellfish (servings/week)**	1.98 ± 3.24	1.92 ± 3.08	0.79
**Other types of seafood (servings/week)**	1.12 ± 1.86	1.36 ± 2.32	0.94
**Caloric intake (day)**			
**All**			
From foods other than SSBs	1660.2 ± 352.2	1733.9 ± 386.6	0.07
From SSBs	141.4 ± 225.2	327.2 ± 440.0	<0.01 *
Total calories	1801.6 ± 448.3	2061.1 ± 689.7	<0.01 *
**Girls**			
From foods other than SSBs	1679.2 ± 392.8	1776.1 ± 415.5	0.27
From SSBs	118.6 ± 208.9	488.9 ± 592.9	<0.01 *
Total calories	1797.7 ± 482.0	2265.0 ± 897.5	0.04 *
**Boys**			
From foods other than SSBs	1646.1 ± 320.1	1724.7 ± 382.1	0.16
From SSBs	158.4 ± 236.9	292.1 ± 395.2	0.02 *
Total calories	1804.5 ± 424.1	2016.9 ± 634.1	0.01 *
**Ingested sugar from SSBs (g/week)**			
	117.3 ± 198.1	296.7 ± 419.5	<0.01 *

* p < 0.05.

**Table 3:** Crude and adjusted odds ratios for the association between sweetened beverage consumption and ADHD (*N* = 260).

Variables	Crude		Adjusted ^1^	
	OR (95% CI)	*p* Value	OR (95% CI)	*p* Value
**(A) All subjects**		<0.01 *		0.02 *
0	Reference		Reference	
1–6	1.48 (0.79–2.78)		1.36 (0.61–3.05)	
≥7	4.66 (2.15–10.09)		3.69 (1.291–10.60)	
**(B) Boys only**		0.01 *		0.06
0	Reference		Reference	
1–6	1.33 (0.65–2.73)		1.05 (0.43–2.59)	
≥7	3.61 (1.47–8.88)		3.54 (1.057–11.95)	

**^1^** Adjusted covariates: gender, consumption of milk/meat/fruit/vegetables, family history of nervous system diseases, parental education levels, maternal alcohol consumption during pregnancy, gene polymorphism of DRD4 at rs752306; * *p* < 0.05.

## Supplementary Materials

The following materials are available online at www.mdpi.com/1660-4601/13/7/678/s1, Figure S1: Boxplot diagram of BLLs for ADHD and normal control participants, Table S1: Relationship between maternal education level and smoking/alcohol consumption during pregnancy, Table S2: Polymorphism of dopamine-related genes (DRD4/DAT1) of the study participants, Table S3: Frequently used artificial food colorings (AFCs) and estimated dosage, Table S4: Frequently used preservatives and estimated maximum exposure.

## Abbreviations

The following abbreviations are used in this manuscript:

ADHD	Attention deficit/hyperactivity disorder
SSB	Sugar-sweetened beverage
DRD4	D4 dopamine receptor gene
DAT1	Dopamine transporter gene
AFC	Artificial food coloring
DSM-IV-TR	Diagnostic and statistical manual of mental disorders, 4th edition, revised criteria
BLL	Blood lead levels
SNAP-IV	Swanson, Nolan and Pelham, fourth revision
MOHW	Ministry of Health and Welfare
TFDA	Taiwan Food and Drug Administration
LOD	Limit of detection
MC	Monte Carlo
OR	Odds ratio
CI	Confidence interval
ADI	Acceptable daily intake
MOS	Margin of safety
BA	Benzoic acid
SA	Sorbic acid
WHO	World Health Organization
