# Supplementary material for: Sugar-Sweetened Beverage Consumption Is Adversely Associated with Childhood Attention Deficit/Hyperactivity Disorder
Source: Int J Environ Res Public Health. 2016 Jul 4;13(7):678. doi: 10.3390/ijerph13070678 (PMC4962219; doi:10.3390/ijerph13070678)
Supplement: Supplementary file 1 [file ijerph-13-00678-s001.pdf]

Supplementary Materials: Sugar-Sweetened Beverage Consumption Is Adversely Associated with Childhood Attention Deficit/Hyperactivity Disorder

Ching-Jung Yu, Jung-Chieh Du, Hsien-Chih Chiou, Chun-Cheng Feng, Ming-Yi Chung, Winnie Yang, Ying-Sheue Chen, Ling-Chu Chien, Betau Hwang and Mei-Lien Chen

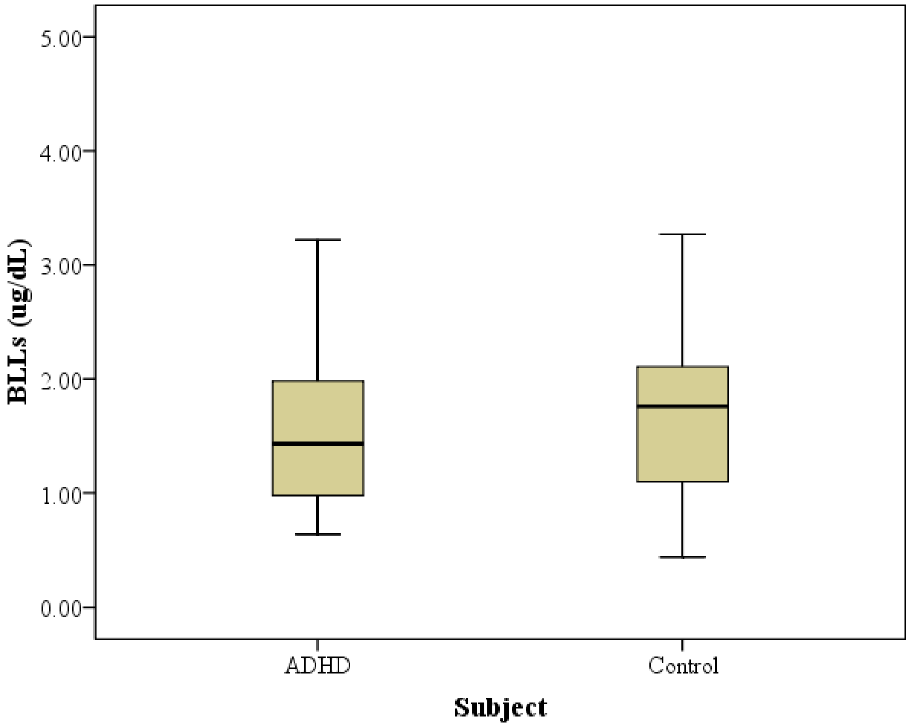

Figure S1. Boxplot diagram of BLLs for ADHD and normal control participants.

Table S1. Relationship between maternal education level and smoking/alcohol consumption during pregnancy.

Variables	Maternal Education		*p*-Value
	**High School and Below (*N* = 139)**	**College or Advanced Training (*N* = 193)**	
**Smoking during pregnancy (%)**			0.02 *
No	123 (88.5%)	187 (96.9%)	
Yes	16 (11.5%)	6 (3.1%)	
**Alcohol consumption during pregnancy (%)**			0.06
No	122 (87.8%)	181 (93.8%)	
Yes	17 (12.2%)	12 (6.2%)	

* $p < 0.05$.

Table S2. Polymorphism of dopamine-related genes (DRD4/DAT1) of the study participants ($N = 207$).

Marker Name	Controls	ADHD
	$N = 110$	$N = 97$
**Gene polymorphism—DRD4**		
**rs7395429**		
T	53 (48.2%)	60 (61.9%)
TC	46 (41.8%)	30 (30.9%)
C	11 (10.0%)	7 (7.2%)
**rs3758653**		
T	44 (40.0%)	48 (49.5%)
TC	55 (50.0%)	45 (46.4%)
C	11 (10.0%)	4 (4.1%)
**rs11246228**		
T	34 (30.9%)	18 (18.6%)
TC	51 (46.4%)	50 (51.5%)
C	25 (22.7%)	29 (29.9%)
**rs752306** [1,*]		
G	67 (60.9%)	75 (77.3%)
GA	38 (34.5 %)	21 (21.6%)
A	5 (4.5%)	1 (1.0%)
**Gene polymorphism—DAT1**		
**rs6347**		
G	1 (0.9%)	0 (0%)
GA	28 (25.5%)	19 (19.6%)
A	81 (73.6%)	78 (80.4%)
**rs2975292**		
G	87 (79.1%)	70 (72.2%)
CG	22 (20.0%)	27 (27.8%)
C	1 (0.9%)	0 (0.0%)
**rs37022**		
A	25 (22.7%)	18 (18.6%)
TA	55 (50.0%)	63 (64.9%)
T	30 (27.3%)	16 (16.5%)
**rs40358**		
T	46 (41.8%)	37 (38.1%)
GT	48 (43.6%)	50 (51.5%)
G	16 (14.3%)	10 (10.3%)
**rs10040882**		
T	1 (0.9%)	0 (0.0%)
TC	22 (20.0%)	24 (24.7%)
C	87 (79.1%)	73 (75.3%)
**rs464049**		
T	16 (14.5%)	7 (7.2%)
TC	44 (40.0%)	47 (48.5%)
C	50 (45.5%)	43 (44.3%)

[1] The significant difference in gene polymorphisms (rs752306) may have resulted from the small size of participants; * $p < 0.05$.

Table S3. Frequently used artificial food colorings (AFCs) and estimated dosage.

Category	ADI [1] (mg/day/kg b.w.)	Max. Permitted Conc. in Beverage (mg/kg)	95th Percentile Exposure		MOS [2]
			Groups	Estimated Max. AFCs Consumed (mg/day/kg b.w.)	
Allura Red	7	100	ADHD	1.93	3.63
			Control	0.93	7.53
Tartrazine	7.5	100	ADHD	1.93	3.89
			Control	0.93	8.06
Sunset Yellow	2.5	100	ADHD	1.93	1.30
			Control	0.93	2.69

[1] ADI: Acceptable daily intake (mg/kg of body weight, based on a standard child with body weight of 30 kg)—estimated amount that children can ingest daily "without appreciable risk"; [2] Margin of safety (ADI divided by the 95th percentile daily AFC consumption through SSBs).

Table S4. Frequently used preservatives and estimated maximum exposure.

Category	ADI [1] (mg/day/kg b.w.)	Max. Permitted Conc. in Beverage (mg/kg)	95th Percentile Exposure		MOS [2]
			Groups	Estimated Max. Preservatives Consumed (mg/day/kg b.w.)	
Benzoic acid	5	1000	ADHD	62.40	0.08
			Control	31.54	0.16
Sorbic acid	25	1000	ADHD	62.40	0.40
			Control	31.54	0.79

[1] ADI: Acceptable daily intake (mg/kg of body weight, based on a standard child with body weight of 30 kg)—estimated amount that children can ingest daily "without appreciable risk"; [2] MOS: Margin of safety (ADI divided by the 95th percentile daily preservative consumption through SSBs).

© 2016 by the authors. Submitted for possible open access publication under the terms and conditions of the Creative Commons Attribution (CC-BY) license (http://creativecommons.org/licenses/by/4.0/).
